# Development and validation of a blood routine-based extent and severity clinical decision support tool for ulcerative colitis

**DOI:** 10.1038/s41598-023-48569-5

**Published:** 2023-12-04

**Authors:** Hongliang Chen, Xindi Lin, Xinyue Pan, Hongyu Xu, Xuemei Zhang, Guoying Liang, Jiawei Qiu, Xueyan Zhang, Yang Gao, Xin Tan, Ning Li, Huimin Cai, Xueyu Cang, Jihan Qi, Wei Li, Shuang Li, Yutong Zheng, Lei Zhao, Shizhu Jin

**Affiliations:** 1 Department of Gastroenterology and Hepatology, The Second Affiliated Hospital of Harbin Medical University, 246 Xuefu Road, Nangang District, Harbin, 150086 Heilongjiang China; 2 Department of Statistics, University of Wisconsin, Madison, WI USA; 3 School of Economics and Finance, Xi’an Jiaotong University, Xi’an, China; 4 Department of Gastroenterology and Hepatology, The First Affiliated Hospital of Harbin Medical University, Harbin, China; 5 Department of Gastroenterology, The First Affiliated Hospital of Jiamusi University, Jiamusi, China; 6 Department of Liver, Spleen and Stomach Diseases, The First Affiliated Hospital of Heilongjiang University of Chinese Medicine, Harbin, China

**Keywords:** Inflammatory bowel disease, Diagnostic markers

## Abstract

Monitoring extent and severity is vital in the ulcerative colitis (UC) follow-up, however, current assessment is complex and low cost-effectiveness. We aimed to develop a routine blood-based clinical decision support tool, Jin’s model, to investigate the extent and severity of UC. The multicentre retrospective cohort study recruited 975 adult UC inpatients and sub-grouped into training, internal validation and external validation set. Model was developed by logistics regression for the extent via Montreal classification and for the severity via Mayo score, Truelove and Witts score (TWS), Mayo endoscopic score (MES) and Degree of Ulcerative colitis Burden of Luminal Inflammation (DUBLIN) score. In Montreal classification, left-sided and extensive versus proctitis model achieved area under the receiver operating characteristic curve (AUROC) of 0.78 and 0.81 retrospectively. For severity, Mayo score model, TWS model, MES model and DUBLIN score model achieved an AUROC of 0.81, 0.70, 0.74 and 0.70 retrospectively. The models also were evaluated with satisfactory calibration and clinical unity. Jin’s model was free with open access at http://jinmodel.com:3000/. Jin’s model is a noninvasive, convenient, and efficient approach to assess the extent and severity of UC.

## Introduction

Ulcerative colitis (UC) has rapidly increased in incidence and prevalence worldwide^1^, especially in newly industrialized countries, including China^2^. UC patients often experience periods of remission and recurrence that cannot be completely avoided^3^. Secular monitoring is beneficial for resolving mucosal inflammation to prevent disease complications such as toxic megacolon, primary sclerosing cholangitis, and risk of colon cancer, which is vital in UC management^3,4^.

Extent and severity assessment is important in UC diagnosis^3^. Montreal classification is used for extent, which is essential for the route of administration. Suppositories and enemas are inclined to proctitis, and intravenous injection and oral administration are given priority for extensive colitis^5,6^. In addition, extensive colitis has a higher risk of colectomy than procotitis^7^. Scoring systems such as the Truelove & Witts score (TWS), Mayo score, Mayo endoscopic score (MES) and Degree of Ulcerative colitis Burden of Luminal Inflammation (DUBLIN) score are often used by clinicians to determine UC severity, which is necessary for drug regimens and doses^3,5,8^. Treatment with aminosalicylates is safe and efficient for mild patients, while systemic corticosteroids and antitumour necrosis factor agents are preferred for moderate and severe patients^5,9^. Endoscopic remission is presently considered the goal of treatment, which is timing for adjusting the therapeutic schedule. Nevertheless, clinical manifestations, laboratory examinations, and colonoscopy are necessary for the above scoring systems, which require a considerable amount of cost effectiveness^3,5,10^. Among them, colonoscopy, regarded as the gold standard, provides objective and explicit proof to evaluate UC^4,11^, which is not suitable for repeated follow-up for each patient in terms of its invasiveness, exorbitant price, poor tolerance, and time consumption. Therefore, supposed simple surrogate markers capable of completing monitoring assessments will be beneficial to simplify the follow-up process, reduce the finical and psychological burden of patients and rationalize the allocation of medical resources.

Previous studies found that UC patients had characteristics of leukocytosis, thrombocytosis, and anaemia in peripheral blood^12,13^, the reason for which is that peripheral blood cells participate in the occurrence and development of UC. Leukocytes and platelets affect each other, exert synergistic effects, and participate in epithelial barrier dysfunction and disorders of intrinsic and extrinsic coagulation^14,15^. However, the evaluation value of routine blood tests in UC has not been systematically elucidated to date.

This study aimed to develop a routine blood-based clinical decision support tool for the extent and severity of UC, providing a simple and practical approach for UC assessment.

## Methods

### Study population

A total of 2015 UC inpatients between January 2010 and December 2019 at the Department of Gastroenterology and Hepatology 4 medical centres. The Training set and internal validation set was based on the data in Second Affiliated Hospital of Harbin Medical University. The external validation set was based on the data in The First Affiliated Hospital of Harbin Medical University, The First Affiliated Hospital of Jiamusi University, and The First Affiliated Hospital of Heilongjiang University of Chinese Medicine.

**Figure 1:**
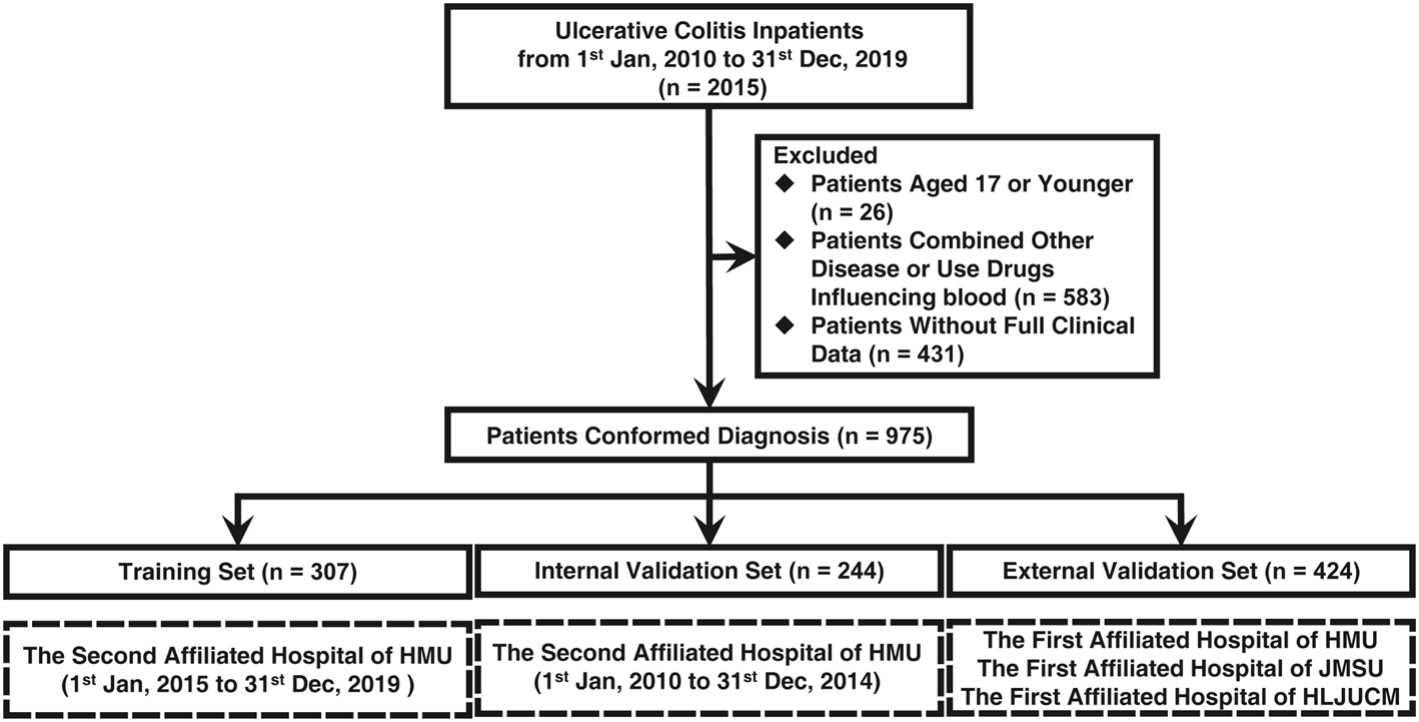
Flow chart of the study population. *HMU* Harbin medical university, *JMSU* Jiamusi University, *JUCM* Heilongjiang University of Chinese Medicine.

We excluded patients with 17 years of age or younger, incomplete clinical data, associated with other inflammatory diseases, associated with benign or malignant tumours or severe organ dysfunction; and associated with haematological diseases or use of drugs that affect blood coagulation function during the past three months. Therefore, the remaining 975 inpatients (307 for training set, 244 for internal validation set and 424 for external validation set) were recruited for the study (Fig. 1). A flow chart of the study population in each study centre is shown in Fig. S1.

### Colonoscopy examination

UC patients took polyethene glycol electrolyte powder for bowel preparation before the colonoscopy examination. Colonoscopy was performed using devices (H260 and H290, Olympus Medical Systems, Tokyo, Japan) by experienced gastroenterologists from each centre.

### UC evaluation

Montreal classification is used to describe UC extent: proctitis (E1), left-sided (E2), and extensive (E3)^3^. The TWS, Mayo score, MES and DUBLIN score are used to describe UC severity. TWS comprises five subscores, including bloody stool/day, pulse, temperature, haemoglobin, erythrocyte sedimentation rate (ESR) and C reactive protein (CRP)^3^. The Mayo score comprises four subscores, including stool frequency, rectal bleeding, mucosa and physician’s global assessment^3^. MES is defined as follows: normal or inactive disease (MES 0), mild (MES 1), moderate (MES 2) and severe (MES 3), in which MES 0 and 1 are defined as endoscopic remission, and MES 2 and 3 are defined as endoscopic activity^5^ (Fig. S2). The DUBLIN score is equal to the product of the MES (0–3) and Montreal classification (E1 to E3). DUBLIN scores ≤ 3 are defined as low inflammation burden, and scores > 3 are defined as high inflammation burden^8^.

### Statistical analysis

Continuous variables were declared as medians with interquartile ranges. Categorical variables were reported using frequencies and percentages. *P* < 0.05 was considered significant.

### Model construction and evaluation

#### Variables selection

Spearman's rank correlation coefficient was used to calculate the correlation among 24 independent variables in routine blood tests. Analysis of variance (ANOVA) was used to determine the significantly different variables. Before the variables were incorporated into the models, collinearity tests were considered to avoid severe overfitting of the models. We considered excluding severely collinear variables according to forward stepwise logistic regression. The elastic net regularization term can automatically select variables in the training process.

#### Model construction

Multivariate logistic regression was used to develop models. When predicting the Montreal classification and DUBLIN score, Youden indexes were used to obtain optimal cut-off values. When predicting Mayo, TWS and MES, the elastic-net penalty and fivefold cross validation were utilized to choose hyperparameters. Polynomial transformation and interaction terms added the nonlinearity of independent variables. Models were trained by the class-weighted loss because of class imbalance (Appendix S1). Sex and age were considered covariates to adjust for potentially confounding factors.

#### Model evaluation

Microaverage was used to evaluate multicategorical models (Appendix S1). Discrimination was assessed using AUROC curves. We used 1000 bootstrap resamplings to reduce the overfit bias. Calibration was assessed using a comparison of predicted probability versus observed probability and mean absolute error (MAE). Clinical unity was assessed using decision curve analysis (DCA) and clinical impact curve (CIC). In addition, we calculated the accuracy, sensitivity, specificity, positive and negative predictive values, positive and negative predictive values, and F1-score to evaluate the models.

### Independent factors analysis

Univariate and multivariate logistic analyses were used to select independent risk and protective factors. Independent variables in each model were enrolled in univariate analysis. The variables with *P* < 0.05 were enrolled in multivariate analysis. The multivariate analysis was adjusted for sex and age.

All data were analysed using Statistical Package for Social Sciences 26.0 (SPSS, Inc. Chicago, Illinois, USA), Python 3.6.5 with the scikit-learn package and R version 4.1.2 (R Foundation for Statistical Computing, Vienna, Austria).

### Statement and ethics

All patients gave informed consent for participation. The study protocol was reviewed and approved by the Ethics Committee of the Second Affiliated Hospital of Harbin Medical University (Ethics review batch number: KY2022-282) and Chinese Clinical Trail Registry (Registration number: ChiCTR2200065388). All procedures performed in studies involving human participants were in accordance with Helsinki declaration. This study is reported as per the Transparent Reporting of a multivariable prediction model for Individual Prognosis or Diagnosis (TRIPOD) guideline (S1 Checklist).

## Results

### Study population

A total of 975 UC patients were included in the study, including 307 in the training set, 244 in the internal validation set, and 424 in the external validation set. Baseline characteristics of enrolled 975 patients are depicted in Table 1 and Table S1.

**Table 1:** Baseline demographic, disease characteristics and complete blood count of ulcerative colitis patients.

Characteristics	Training set	Internal validation set	External validation set
	(n = 307)	(n = 244)	(n = 424)
Demographics			
Age [years, mean (min–max)]	45.68 (18–77)	42.11 (18–76)	49.33 (18–89)
Sex (male/female)	181/126	140/104	228/196
Disease characteristics			
Disease extent [n (%)]			
Proctitis (E1)	33 (10.75)	36 (14.75)	72 (17.45)
Left-sided (E2)	75 (24.43)	76 (31.15)	107 (25.24)
Extensive (E3)	196 (64.84)	128 (52.46)	243 (57.31)
Unknown	3 (0.98)	4 (1.64)	0 (0.00)
Truelove and Witts score [n (%)]			
Mild	114 (37.13)	109 (44.67)	72 (16.98)
Moderate	110 (35.83)	91 (37.30)	200 (47.17)
Severe	83 (27.04)	44 (18.03)	152 (35.85)
Mayo classification [n (%)]			
Clinical remission or mild	104 (33.88)	91 (37.30)	75 (17.69)
Moderate	165 (53.75)	128 (52.46)	278 (65.57)
Severe	38 (12.38)	25 (10.25)	71 (16.75)
Mayo endoscopic score [n (%)]			
Endoscopic remission	79 (25.73)	73 (29.92)	36 (8.49)
Endoscopic activity	228 (74.27)	171 (70.08)	388 (91.51)
DUBLIN score [n (%)]			
Low inflammation burden	99 (32.25)	91 (37.30)	54 (12.74)
High inflammation burden	208 (67.75)	153 (62.70)	370 (87.26)

*BASO* basophil, *CV* coefficient of variation, *DUBLIN* degree of ulcerative colitis burden of luminal inflammation, *EO* eosinophil, *HCT* haematocrit, *HGB* haemoglobin, *IQR* interquartile range, *LYMPH* lymphocyte, *MCH* mean corpuscular haemoglobin, *MCHC* mean corpuscular hemoglobin concentration, *MCV* mean corpuscular volume, *MONO* monocyte, *MPV* mean platelet volume, *NEUT* neutrophil, *PCT* thrombocytocrit, *PDW* platelet distribution width, *P-LCR* platelet large cell ratio, *PLT* platelet, *RBC* red blood cell, *RDW* red cell distribution width, *SD* standard deviation, *WBC* white blood cell.

### Development and evaluation of Jin’s model

**Figure 2:**
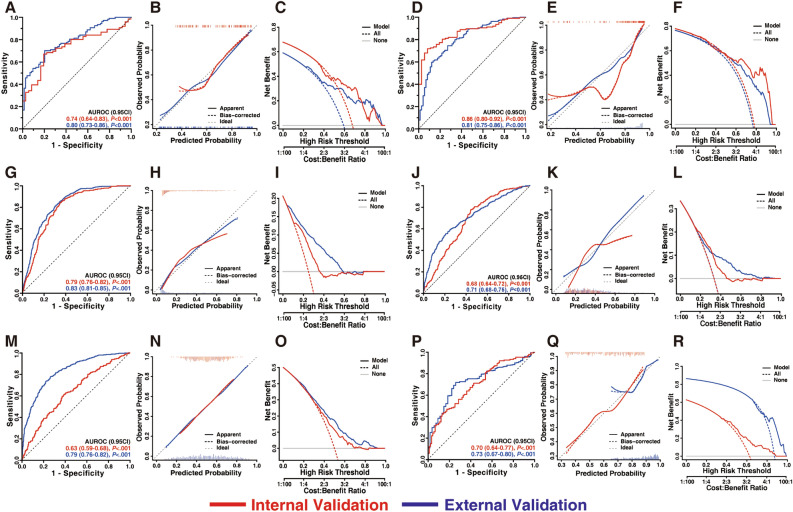
The evaluation of models for UC extent (**A**–**F**) and severity (**G**–**R**). (**A**–**C**) present model for distinguishing E2 from E1. (**D**–**F**) present model for distinguishing E3 from E1. (**G**–**I**) present model for predicting Mayo score. (**J**–**L**) present model for predicting TWS. (**M**–**O**) present model for predicting MES. (**P**–**R**) present model for predicting DUBLIN score. (**A**,**D**,**G**,**J**,**M**,**P**) ROC curves. (**B**,**E**,**H**,**K**,**N**,**Q**) Calibration curves. Smoothed lines fit to the curve and vertical bar illustrates the distribution of predictions. (**C**,**F**,**I**,**L**,**O**,**R**) Decision curves. Red and blue lines represent internal and external validation. Abbreviation: AUROC, area under the receiver operating characteristic.

We constructed and validated six prediction models and named Jin’s model. Physicians and UC patients can use Jin’s model freely at http://jinmodel.com:3000/. The ANOVA of routine blood tests in the scoring systems is shown in Table S2. The model was adjusted for sex and age (Table S3). The model evaluation is shown in Fig. 2 and Table 2. More details are shown in Appendices S1 and S2.

**Table 2:** Model performance of Jin’s model.

Variables		Accuracy	Sensitivity	Specificity	PPV	NPV	PLR	NLR	F1-score
Montreal classification									
E2 vs. E1	Internal validation	0.65	0.58	0.81	0.86	0.48	2.98	0.52	0.83
	External validation	0.68	0.56	0.85	0.85	0.57	3.77	0.52	0.85
E3 vs. E1	Internal validation	0.79	0.83	0.64	0.89	0.51	2.30	0.27	0.74
	External validation	0.74	0.76	0.69	0.89	0.46	2.43	0.35	0.77
Severity									
Mayo score*	Internal validation	0.70	0.55	0.77	0.55	0.78	2.48	0.58	0.65
	External validation	0.71	0.57	0.79	0.57	0.78	2.63	0.55	0.66
TWS*	Internal validation	0.65	0.46	0.74	0.48	0.73	1.80	0.73	0.58
	External validation	0.73	0.59	0.79	0.59	0.79	2.87	0.52	0.68
MES	Internal validation	0.61	0.66	0.52	0.76	0.39	1.37	0.66	0.62
	External validation	0.67	0.69	0.50	0.94	0.13	1.38	0.62	0.65
DUBLIN score	Internal validation	0.65	0.71	0.55	0.73	0.53	1.58	0.52	0.63
	External validation	0.68	0.67	0.76	0.95	0.25	2.80	0.43	0.84

E2 vs. E1 presents distinguish left-sided from proctitis, E3 vs. E1 presents distinguish extensive from proctitis.

*DUBLIN* degree of ulcerative colitis burden of luminal inflammation, *MES* Mayo endoscopic score, *NLR* negative likelihood ratio, *NPV* negative predictive value, *PLR* positive likelihood ratio, *PPV* positive predictive value, *TWS* Truelove and Witts score.

*The multi-categorical models were used micro-average.

### Establishment of models for UC extent

Because no validated independent variables in routine blood tests were found to distinguish E2 from E3, we constructed two separate models for distinguishing E2 from E1 and E3 from E1.

The prediction values in E2 were significantly higher than those in E1 (median [interquartile range, (IQR)], internal validation 0.74 [0.62–0.83] vs. 0.60 [0.56–0.67], *P* < 0.001; external validation 0.78 [0.63–0.91] vs. 0.59 [0.53–0.66], *P* < 0.001). The model had an AUROC of 0.74 (95% CI 0.64–0.83, *P* < 0.001) in internal validation and 0.81 (95% CI 0.75–0.87, *P* < 0.001) in external validation (Fig. 2A), and an MAE of 0.021 in internal validation and 0.018 in external validation (Fig. 2B). When an optimal cut-off value of 0.72 was applied, DCA (Fig. 2C) was performed with a standard net benefit (sNB) of 0.42 in internal validation and 0.34 in external validation.

The prediction values in E3 were significantly higher than those in E1 (internal validation 0.94 [0.87–0.97] vs. 0.78 [0.68–0.85], *P* < 0.001; external validation 0.93 [0.85–0.97] vs. 0.76 [0.62–0.86], *P* < 0.001). Model 2 had an AUROC of 0.86 (95% CI 0.80–0.92, *P* < 0.001) in internal validation and 0.81 (95% CI 0.75–0.86, *P* < 0.001) in external validation (Fig. 2D), an MAE of 0.074 in internal validation and 0.028 in external validation (Fig. 2E). When an optimal cut-off value of 0.84 was applied, DCA (Fig. 2F) was performed with an sNB of 0.60 in internal validation and 0.34 in external validation.

To output a definite classification, we combined two models and summarized four possible results and their diagnostic adjudications (Table S4).

### Establishment of models for UC severity

#### Establishment of a model for predicting Mayo score

**Figure 3:**
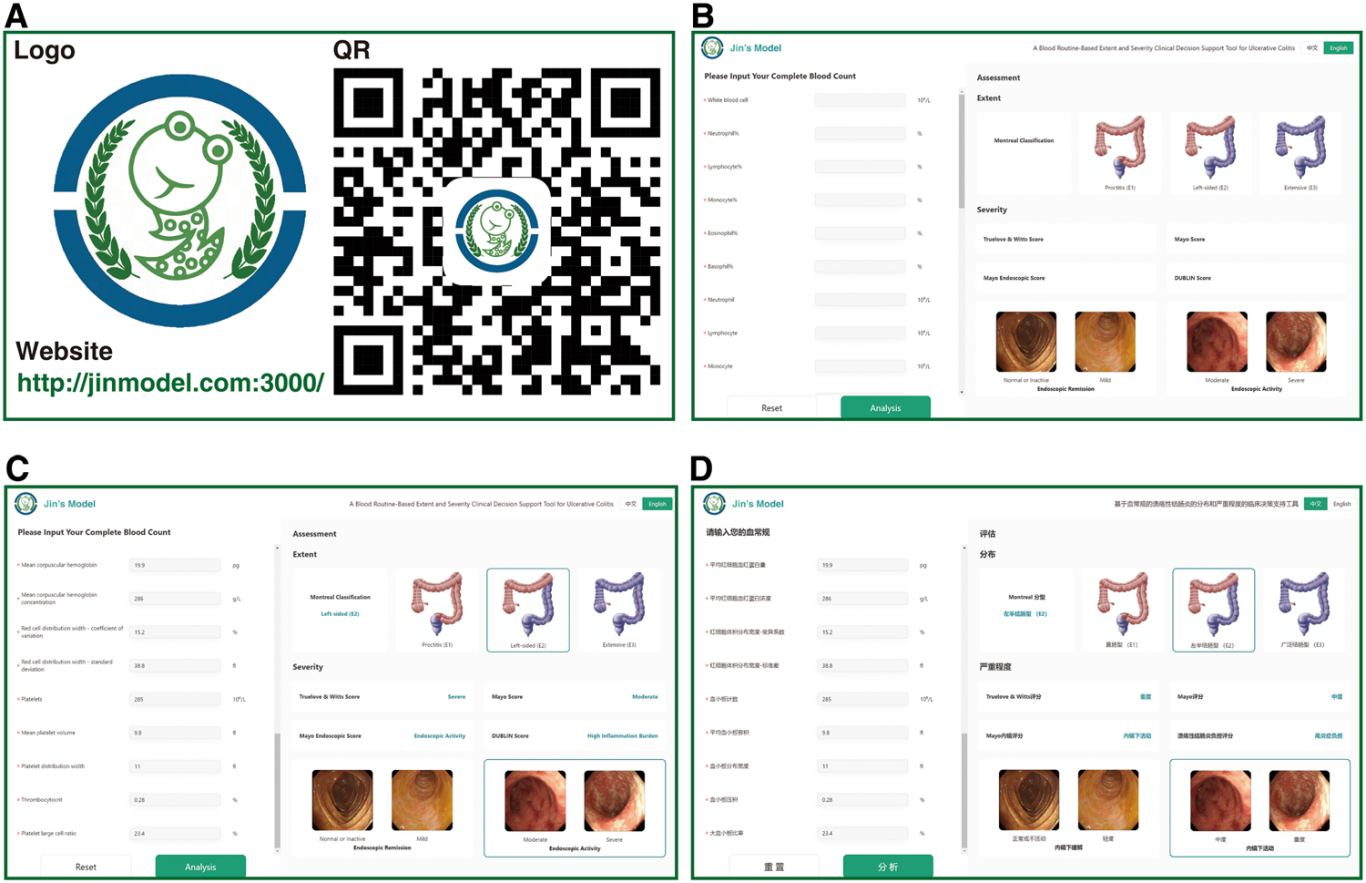
Online Jin’s model: http://jinmodel.com:3000/. (**A**) The logo, Website and QR of Jin’ model. (**B**) The presentation of online Jin’s model. (**C**) The website outputs model predictions online in English. (**D**) The website outputs model predictions online in Chinese. *QR* quick response.

The model had an AUROC of 0.79 (95% CI 0.76–0.82, *P* < 0.001) in internal validation and 0.83 (95% CI 0.81–0.85, *P* < 0.001) in external validation (Fig. 3G), and an MAE of 0.037 in internal validation and 0.022 in external validation (Fig. 3H). When an optimal cut-off value of 0.30 was applied, DCA (Fig. 3I) was performed with an sNB of 0.33 in internal validation and 0.44 in external validation.

#### Establishment of a model for predicting TWS

The model had an AUROC of 0.68 (95% CI 0.64–0.72, *P* < 0.001) in internal validation and 0.71 (95% CI 0.68–0.75, *P* < 0.001) in external validation (Fig. 3J), an MAE of 0.054 in internal validation and 0.029 in external validation (Fig. 3K). When an optimal cut-off value of 0.33 was applied, DCA (Fig. 3L) was performed with an sNB of 0.27 in internal validation and 0.32 in external validation.

#### Establishment of a model for predicting MES

The model had an AUROC of 0.63 (95% CI 0.59–0.68, *P* < 0.001) in internal validation and 0.83 (95% CI 0.80–0.85, *P* < 0.001) in external validation (Fig. 3M), and an MAE of 0.004 in internal validation and 0.005 in external validation (Fig. 3N). When an optimal cut-off value of 0.50 was applied, DCA (Fig. 3O) was performed with an sNB of 0.23 in internal validation and 0.34 in external validation.

#### Establishment of a model for predicting DUBLIN score

The model had an AUROC of 0.69 (95% CI 0.62–0.75, *P* < 0.001) in internal validation and 0.73 (95% CI 0.66–0.80, *P* < 0.001) in external validation (Fig. 3P), and an MAE of 0.025 in internal validation and 0.021 in external validation (Fig. 3Q). When an optimal cut-off value of 0.67 was applied, DCA (Fig. 2R) was performed with an sNB of 0.15 in internal validation and 0.70 in external validation.

Univariate and multivariate analyses of independent factors in Jin’s model.

#### Montreal classification

WBC (OR [0.95 CI], internal validation: 1.310 [1.061–1.617], *P* = 0.012; external validation: 1.711 [1.376–2.128], *P* < 0.001) and RDW-CV (OR [0.95 CI], internal validation: 1.481 [1.012–2.168], *P* = 0.043; external validation: 2.219 [1.486–3.314], *P* < 0.001) was an independent risk factor (Table 3, Table S5).

**Table 3:** Multivariate analysis adjusting gender and age of independent factors in Jin’s model.

		Internal validation set			External validation set		
		OR	0.95 CI	Adjusted P	OR	0.95 CI	Adjusted P
Montreal classification							
E2 vs. E1							
WBC		1.310	1.061–1.617	0.012	1.711	1.376–2.128	< 0.001
RDW-CV		1.481	1.012–2.168	0.043	2.219	1.486–3.314	< 0.001
E3 vs. E1							
LYMPH%		0.933	0.888–0.981	0.007	0.922	0.890–0.955	< 0.001
EO		82.632	1.644–4152.692	0.027	395.343	18.531–8434.143	< 0.001
Severity							
Mayo score							
WBC	Remission/mild	1					
	Moderate	1.175	1.046–1.319	0.006	1.364	1.196–1.566	< 0.001
	Severe	1.571	1.321–1.869	< 0.001	1.446	1.253–1.669	< 0.001
HCT	Remission/mild	1					
	Moderate	0.875	0.820–0.934	< 0.001	0.944	0.898–0.993	0.026
	Severe	0.725	0.646–0.813	< 0.001	0.860	0.810–0.914	< 0.001
Mayo endoscopic score							
NEUT				0.140	1.200	1.023–1.047	0.025
HCT		0.902	0.847–0.960	0.001	0.904	0.843–0.971	0.005
DUBLIN score							
WBC		1.149	1.039–1.271	0.007	1.668	1.384–2.011	< 0.001
RBC		0.274	0.146–0.512	< 0.001	0.437	0.249–0.765	0.004

E2 vs. E1 presents distinguish left-sided from proctitis, E3 vs. E1 presents distinguish extensive from proctitis.

*CI* confidence interval, *CV* coefficient of variation, *DUBLIN* degree of ulcerative colitis burden of luminal inflammation, *HCT* haematocrit, *HGB* haemoglobin, *NEUT* neutrophil, *OR* odds ratio, *RBC* red blood cell, *RDW* red cell distribution width, *WBC* white blood cell.

Lymphocyte% was an independent protective factor (OR [0.95 CI], internal validation: 0.933 [0.888–0.981], *P* = 0.007; external validation: 0.922 [0.890–0.955], *P* < 0.001), and eosinophils were independent risk factors (OR [0.95 CI], internal validation: 82.632 [1.644–4152.692], *P* = 0.027; external validation: 395.343 [18.531–8434.143], *P* < 0.001) (Table 3, Table S6).

#### Mayo score

WBC was an independent risk factor (OR [0.95 CI], internal validation: moderate 1.175 [1.046–1.319], *P* = 0.006, severe 1.571 [1.321–1.869], *P* < 0.001; external validation: moderate 1.364 [1.196–1.566], *P* < 0.001, severe 1.446 [1.253–1.669], *P* < 0.001), and haematocrit was an independent protective factor (OR [0.95 CI], internal validation: moderate 0.875 [0.820–0.934], *P* < 0.001, severe 0.725 [0.646–0.813], *P* < 0.001; external validation: moderate 0.944 [0.898–0.993],* P* = 0.026, severe 0.860 [0.810–0.914], *P* < 0.001) to Mayo classification (Table 3, Table S7).

#### MES

Haematocrit was an independent protective factor (OR [0.95 CI], internal validation: 0.847 [0.894–0.960], *P* = 0.001; external validation: 0.904 [0.843–0.971], *P* = 0.005) (Table 3, Table S8).

#### DUBLIN score

WBC was an independent risk factor (OR [0.95 CI], internal validation: 1.149 [1.039–1.271], *P* = 0.007; external validation: 1.668 [1.384–2.011], *P* < 0.001), and RBC count was an independent protective factor (OR [0.95 CI], internal validation: 0.274 [0.146–0.512], *P* < 0.001; external validation: 0.437 [0.249–0.765], *P* = 0.004) (Table 3, Table S9).

## Discussion

To the best of our knowledge, Jin’s model, composed of two models for predicting Montreal classification and four models for predicting Mayo score, TWS, MES and DUBLIN score, is the first simple clinical support decision tool for evaluating the extent and severity of UC based on routine blood.

**Figure 4:**
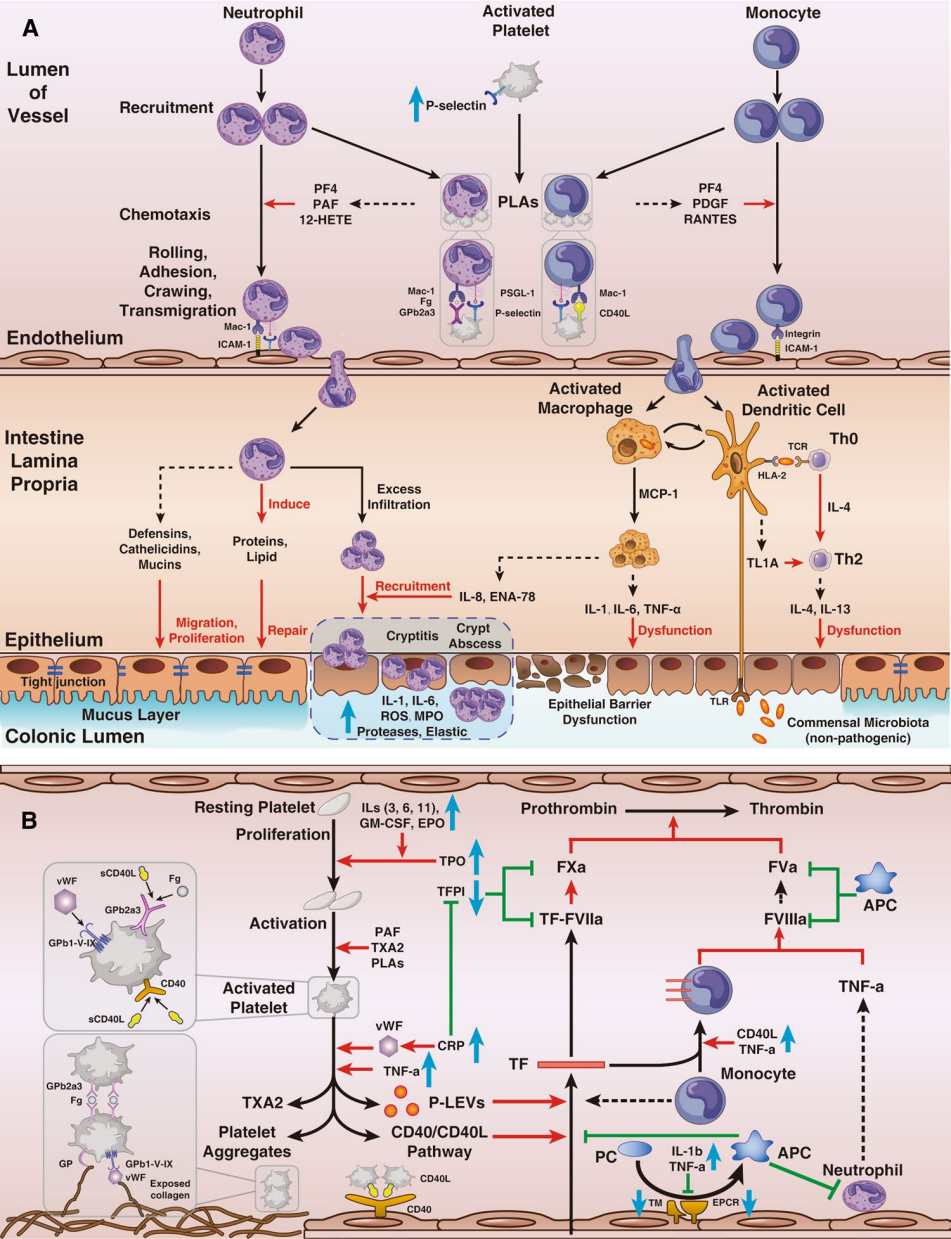
Mechanisms of peripheral blood cells in the pathogenesis of UC. (**A**) Peripheral blood cells enter from blood into the intestine and mediate the inflammatory response to damage the intestinal barrier. (**B**) The activated platelets participated in dysfunction of intrinsic and extrinsic blood coagulation. Solid black arrows represented “conversion to”, dashed black arrows represented “release”, red arrows represented “promotion”, green arrows represented “inhibition”, and blue arrows represented “increase and decrease of substances”. *APC* activated protein C, *CD* cluster of differentiation, *CRP* C reactive protein, *ENA* extractable nuclear antigen, *EPCR* endothelial protein C receptor, *EPCR* endothelial protein C receptor, *EPO* erythropoietin, *Fg* fibrinogen, *GM-CSF* granulocyte–macrophage colony-stimulating factor, *GP* glycoprotein, *HETE* hydroxy eicosatetraenoic acid, *HLA* human leukocyte antigen, *ICAM* intercellular adhesion molecule, *IL* interleukin, *L* ligand, *MAC* membrane attack complex, *MCP* monocyte chemotactic protein, *MPO* myeloperoxidase, *PAF* platelet-activating factor, *PC* protein C, *PDGF* platelet-derived growth factor, *PF* platelet factor, *PLAs* platelet-leukocyte aggregates, *P-LEV* platelet-derived large extracellular vesicle, *PSGL* P-selectin glycoprotein ligand, *RANTES* regulated upon activation, normal T cell expressed and presumably secreted, *ROS* reactive oxygen species, *TCR* T cell receptor, *TF* tissue factor, *TFPI* tissue factor pathway inhibitor, *Th* T helper cell, *TL1A* tumor necrosis factor-like ligand 1, *TLR* toll-like receptor, *TM* thrombomodulin, *TNF* tumor necrosis factor, *TPO* thrombopoietin, *TXA* thromboxane, *UC* ulcerative colitis, *VWF* von Willebrand factor.

We chose peripheral blood cells to construct prediction models because they participate in UC development and progression (Fig. 4). Activated platelets participate in the recruitment and chemotaxis of leukocytes, forming platelet-leukocyte aggregates (PLAs)^16–20^. PLAs contribute not only to the amplification of local inflammation in colonic tissues by promoting neutrophil extravasation but also to the exacerbation of thrombogenicity in systemic vessels^19,21,22^. The migration of leukocytes from blood vessels to intestinal tissue follows the leukocyte-adhesion cascade^20,23,24^.

In the intestinal lamina propria, recruitment and apoptosis defects of neutrophils in the epithelium lead to cryptitis or crypt abscesses through several chemotactic molecules and impact the migration, proliferation and protection of epithelial cells^6,19,25,26^. Macrophages and dendritic cells are activated by recognition of nonpathogenic bacteria through Toll-like receptors^15,24^ and are related to epithelial abnormalities. Both cell types exert cytotoxic functions against epithelial cells, including induction of apoptosis and alteration of the protein composition of tight junctions, which leads to epithelial barrier dysfunction^15,27,28^.

In blood vessels, activated platelets interact with exposed collagens, regulate blood coagulation and increase the tendency for intestinal microinfarction as well as systemic thromboembolism. An increased von Willebrand factor mediates the adhesion of activated platelets by forming platelet aggregates and inducing platelet-endothelial interactions, which are vital in endothelial dysfunction and microvascular thrombosis formation^14^. In addition, platelet-derived large extracellular vesicles (P-LEVs) are stronger than activated platelets in pro-coagulation and function in inflammation and angiogenesis^14^. Activated platelets upregulate the secretion of tissue factor (TF) from exposed collagens through P-LEVs and the cluster of differentiation (CD) 40/CD40 ligand pathway, which contribute to extrinsic coagulation^14,20,29^. In addition, leukocytes not only promote the upregulation of TF but also have positive impacts on intrinsic coagulation^14,20^. Therefore, peripheral blood cells are involved in the development of ulcerative colitis by enhancing the inflammatory response of the intestinal mucosa, disrupting the epithelial mucosal barrier and causing coagulation dysfunction.

During model construction, we tried as many methods as possible and selected the most reasonable, robust and well-performing method. For classification, we attempted support vector machine (SVM), decision tree, random forest, bagging, boost and AdaBoost. For the data preprocessing method, we tried principal component analysis, factor analysis, and max absolute value transformation. The results of the prediction models for Mayo score are shown in Table S10. From these results, logistic regression was chosen as the most robust and well-performing method.

In addition, we faced the challenge of an imbalanced data set, in which clinical remission (0.71–5.33%) was far less than the sample size of moderate remission (52.46–65.57%) in the Mayo score. This may result in our study population being focused on inpatients who always had more serious conditions. The predictive model trained by imbalanced data will be skewed to the majority classes. Therefore, we combined clinical remission and mild into one class and used class-weighted loss to compensate for the influence of imbalanced classes on model performance and achieved an accuracy of 0.70 in internal validation and 0.71 in external validation in the model for predicting Mayo classification. We also compared the model with other popular non-invasive markers, CRP and ESR. The AUROC showed Jin’s model had a better diagnostic performance than CRP and ESR (Fig. S3).

The study still had some limitations. First, the sample size was relatively small. We included four centres in northeast China, which neglected different counties, races, and weather except for the northern temperate zone and several special dietary structures. Second, inevitable multicollinearity existed owing to the correlation among independent variables, although we calculated Spearman's rank correlation coefficient (Fig. S4) and VIF and tried to use ANOVA and the elastic net regularization term to reduce it; however, it cannot be completely avoided. Third, instead of building a predictive model to directly distinguish the Montreal classification, we distinguished it with two binary models. In a following study, we also need to find other noninvasive methods to distinguish between E2 and E3. Last, Jin’s model requires inputting the parameters into the calculator, which makes it somewhat less user friendly.

## Conclusion

Jin’s model provides UC patients with a noninvasive, convenient and efficient approach to assess the extent and severity based on several prevailing classifications, especially for patients who do not tolerate or refuse colonoscopy. Jin’s model can simplify the follow-up process, save healthcare resources and reduce the financial and mental burden on patients. Jin's model is of accessibility in a free with open access through http://jinmodel.com:3000/.

### Supplementary Information

Supplementary Information.

Supplementary Information.

## Data Availability

All data generated or analysed during this study are included in this published article (and its supplementary information files). The corresponding author may share study protocol and data transparency upon reasonable request (drshizhujin@hrbmu.edu.cn).
