# Supplementary material for: Development and validation of a blood routine-based extent and severity clinical decision support tool for ulcerative colitis
Source: Sci Rep. 2023 Dec 4;13:21368. doi: 10.1038/s41598-023-48569-5 (PMC10696009; doi:10.1038/s41598-023-48569-5)
Supplement: Supplementary file 1 — Supplementary Information. [file 41598_2023_48569_MOESM1_ESM.pdf]

Development and Validation of a Blood Routine-Based Extent and Severity Clinical Decision Support Tool for Ulcerative Colitis

Hongliang Chen, Xindi Lin, Xinyue Pan, Hongyu Xu, Xuemei Zhang, Guoying Liang, Jiawei Qiu, Xueyan Zhang, Yang Gao, Xin Tan, Ning Li, Huimin Cai, Xueyu Cang, Jihan Qi, Wei Li, Shuang Li, Yutong Zheng, Lei Zhao and Shizhu Jin*

Supplementary Material

Supplementary Figures

Figure S1. Flow chart of the study population in each study center. (A) The Second Affiliated Hospital of Harbin Medical University. (B) The First Affiliated Hospital of Harbin Medical University. (C) The Affiliated Hospital of Jiamusi University. (D) The First Affiliated Hospital of Heilongjiang University of Chinese Medicine.

Figure S2. Endoscopic and histologic H&E tests in UC and HC. (A) HE image of healthy control. (B) HE image of UC patient. (C) Endoscopic image for MES 0 of UC patient. (D) Endoscopic image for MES 1 of UC patient. (E) Endoscopic image for MES 2 of UC patient. (F) Endoscopic image for MES 3 of UC patient.

Figure S3. Comparison of AUROC among Jin's model, CRP and ESR. (A) E2 vs E1.(A) present model for distinguishing E2 from E1. (B) present model for distinguishing E3 from E1. (C) present model for predicting Mayo score. (D) present model for predicting TWS. (E) present model for predicting MES. (F) present model for predicting DUBLIN score.

Figure S4. Correlations among blood routine were calculated by Spearman's rank correlation analysis. (A) all samples. (B) Training set. (C) Internal validation set. (D) External validation set.

Supplementary Tables

Table S1. The blood routine test of ulcerative colitis patients.

Table S2. Analysis of variance of complete blood count of ulcerative colitis patients in the training set.

Table S3. Jin's model adjusted for gender and age.

Table S4. The illustration of Jin's model for predicting UC extent.

Table S5. Univariate and multivariate analysis of independent factors in Jin's model

for differentiating left-sided UC form proctitis UC.

Table S6. Univariate and multivariate analysis of independent factors in Jin's model for differentiating extensive UC form proctitis UC.

Table S7. Univariate and multivariate analysis of independent factors in Jin's model for Mayo score.

Table S8. Univariate and multivariate analysis of independent factors in Jin's model for Mayo endoscopic score.

Table S9. Univariate and multivariate analysis of independent factors in Jin's model for DUBLIN score.

Table S10. Summary evaluation of prediction model for Mayo score with each method.

APPENDIX

Appendix 1. The details of the construction and evaluation of model

Appendix 2. The description of Jin's model

Figure S1

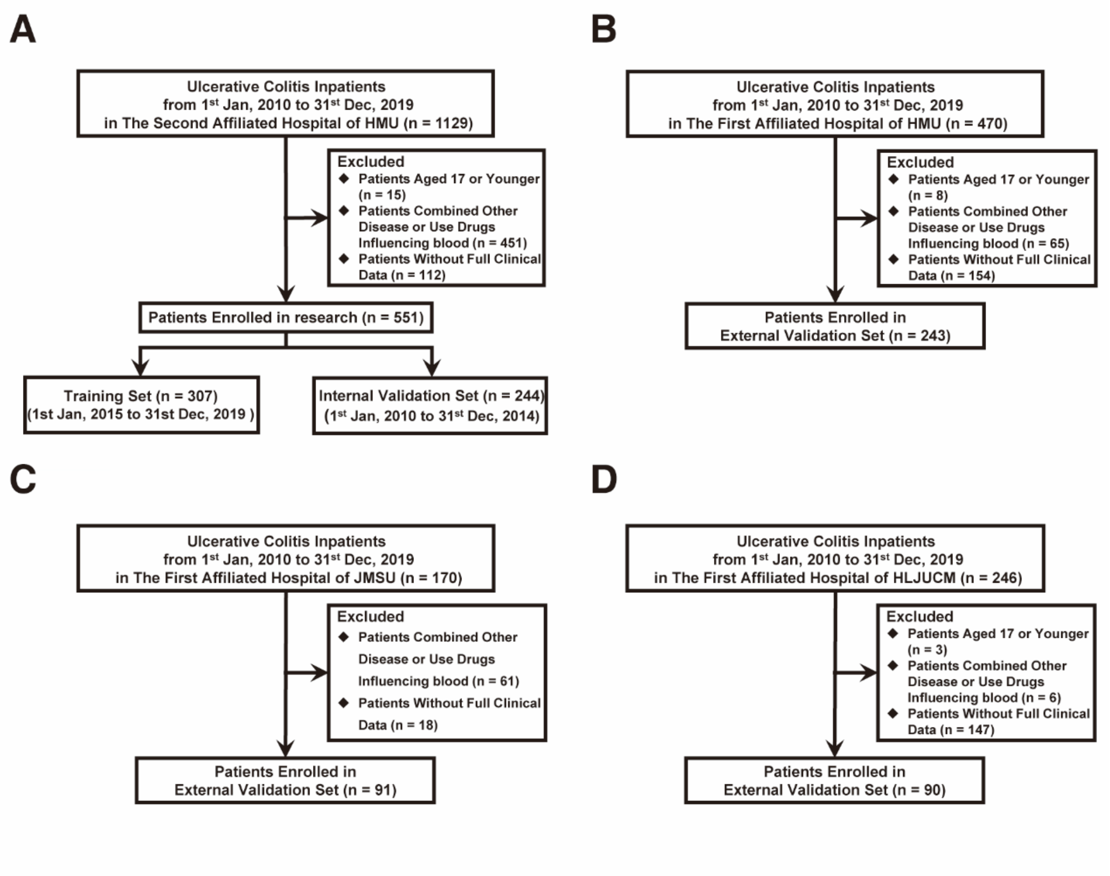
A
Ulcerative Colitis Inpatients from 1st Jan, 2010 to 31st Dec, 2019 in The Second Affiliated Hospital of HMU (n = 1129)
Excluded
Patients Aged 17 or Younger (n = 15)
Patients Combined Other Disease or Use Drugs Influencing blood (n = 451)
Patients Without Full Clinical Data (n = 112)
Patients Enrolled in research (n = 551)
Training Set (n = 307) (1st Jan, 2015 to 31st Dec, 2019)
Internal Validation Set (n = 244) (1st Jan, 2010 to 31st Dec, 2014)
B
Ulcerative Colitis Inpatients from 1st Jan, 2010 to 31st Dec, 2019 in The First Affiliated Hospital of HMU (n = 470)
Excluded
Patients Aged 17 or Younger (n = 8)
Patients Combined Other Disease or Use Drugs Influencing blood (n = 65)
Patients Without Full Clinical Data (n = 154)
Patients Enrolled in External Validation Set (n = 243)
C
Ulcerative Colitis Inpatients from 1st Jan, 2010 to 31st Dec, 2019 in The First Affiliated Hospital of JMSU (n = 170)
Excluded
Patients Combined Other Disease or Use Drugs Influencing blood (n = 61)
Patients Without Full Clinical Data (n = 18)
Patients Enrolled in External Validation Set (n = 91)
D
Ulcerative Colitis Inpatients from 1st Jan, 2010 to 31st Dec, 2019 in The First Affiliated Hospital of HLJUCM (n = 246)
Excluded
Patients Aged 17 or Younger (n = 3)
Patients Combined Other Disease or Use Drugs Influencing blood (n = 6)
Patients Without Full Clinical Data (n = 147)
Patients Enrolled in External Validation Set (n = 90)

Figure S2

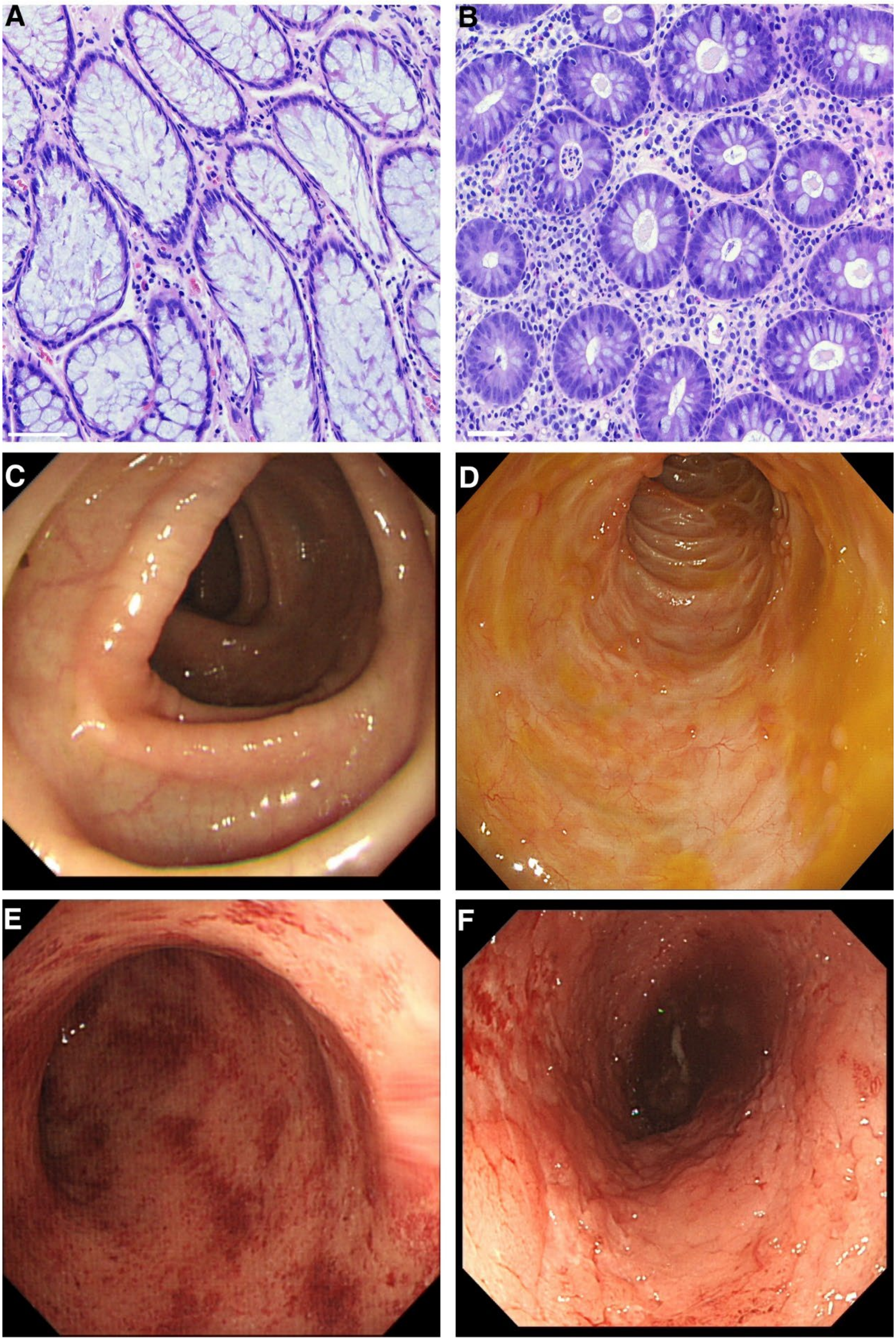

Figure S3

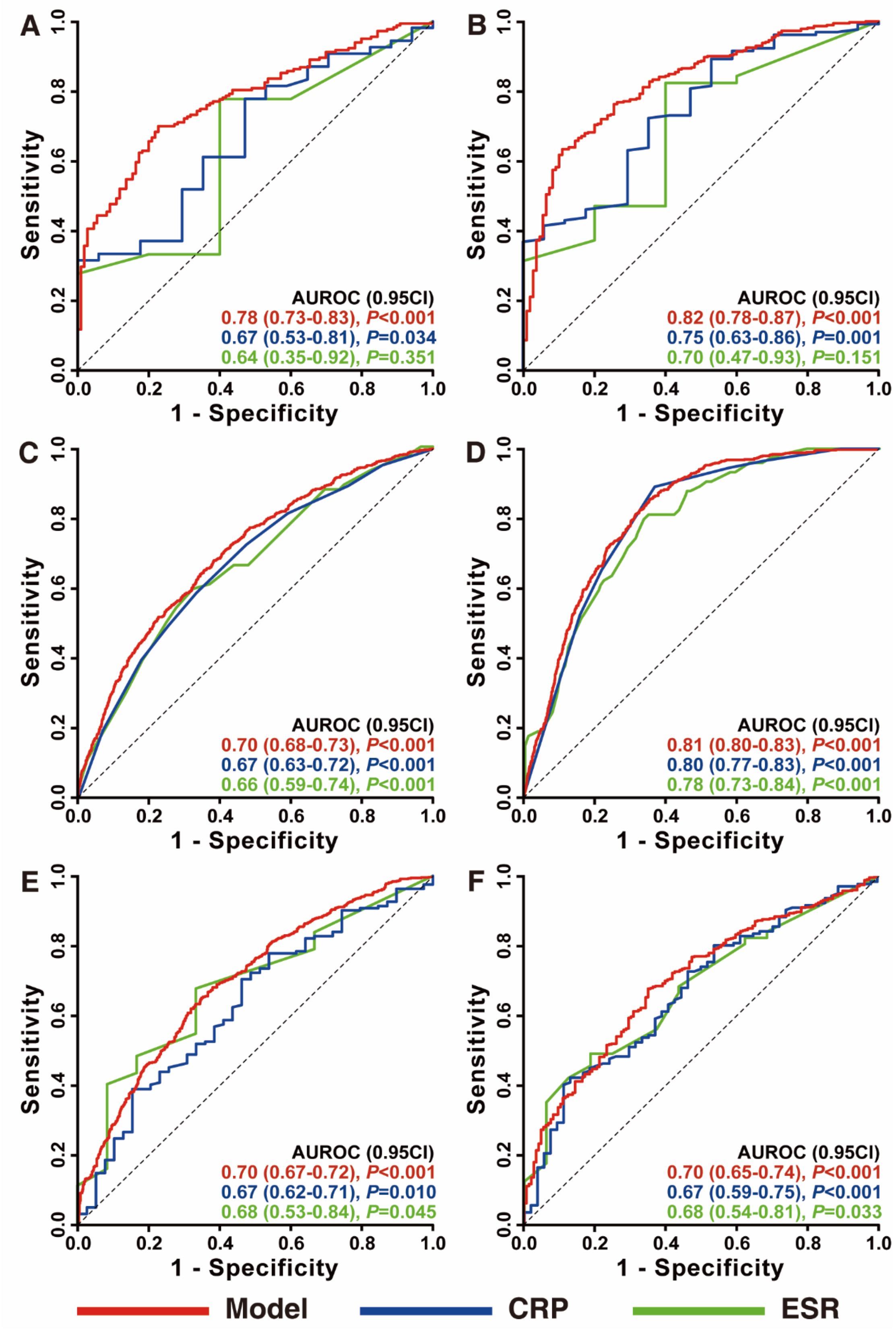

A
B
C
D
E
F
Sensitivity
1 - Specificity
0.0
0.2
0.4
0.6
0.8
1.0
AUROC (0.95CI)
0.78 (0.73-0.83), P<0.001
0.67 (0.53-0.81), P=0.034
0.64 (0.35-0.92), P=0.351
AUROC (0.95CI)
0.82 (0.78-0.87), P<0.001
0.75 (0.63-0.86), P=0.001
0.70 (0.47-0.93), P=0.151
AUROC (0.95CI)
0.70 (0.68-0.73), P<0.001
0.67 (0.63-0.72), P<0.001
0.66 (0.59-0.74), P<0.001
AUROC (0.95CI)
0.81 (0.80-0.83), P<0.001
0.80 (0.77-0.83), P<0.001
0.78 (0.73-0.84), P<0.001
AUROC (0.95CI)
0.70 (0.67-0.72), P<0.001
0.67 (0.62-0.71), P=0.010
0.68 (0.53-0.84), P=0.045
AUROC (0.95CI)
0.70 (0.65-0.74), P<0.001
0.67 (0.59-0.75), P<0.001
0.68 (0.54-0.81), P=0.033
Model
CRP
ESR

Figure S4

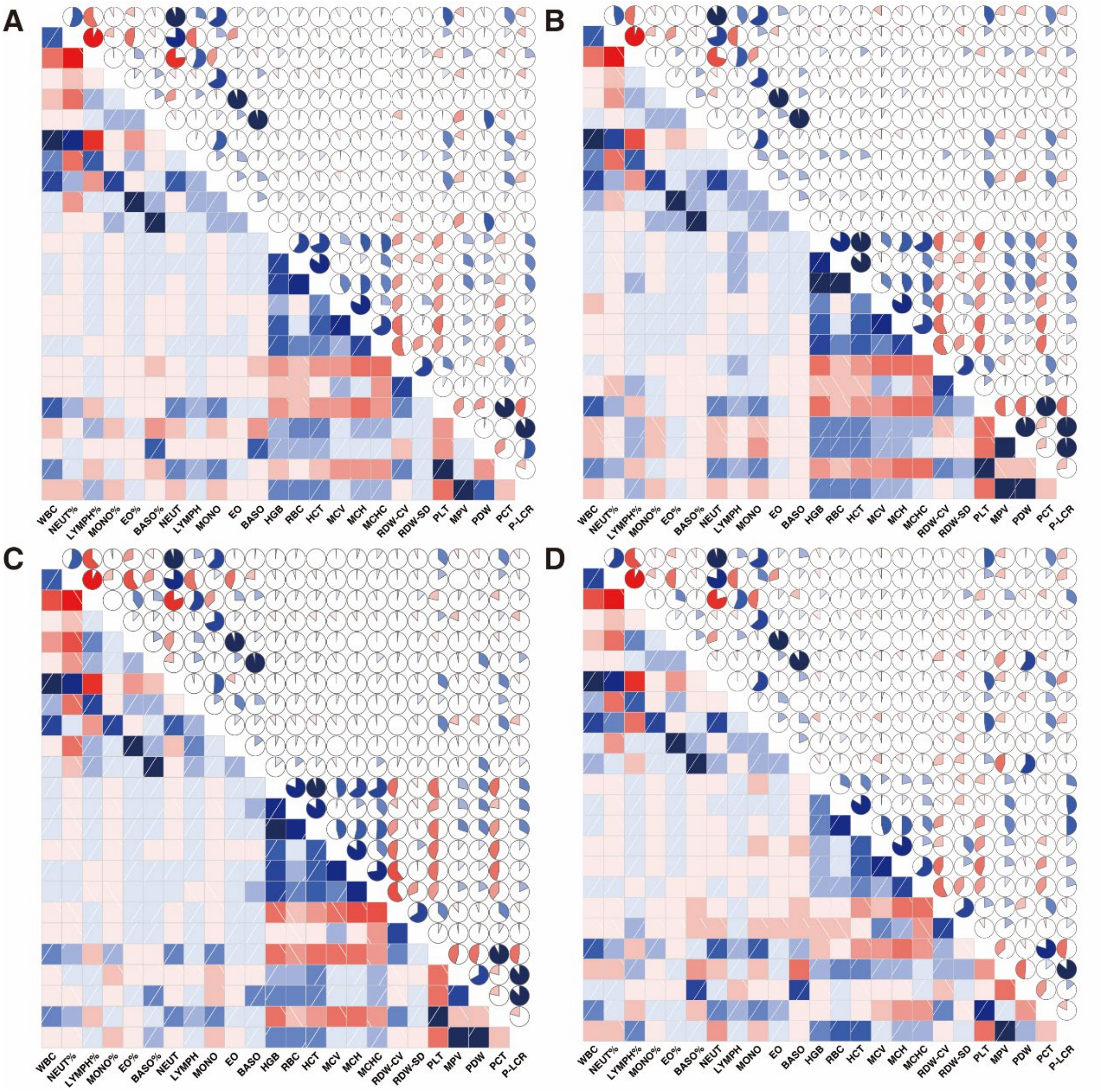
A
B
C
D
WBC
NEUT%
LYMPH%
MONO%
EO%
BASO%
NEUT
LYMPH
MONO
EO
BASO
HGB
RBC
HCT
MCV
MCH
MCHC
RDW-CV
RDW-SD
PLT
MPV
PDW
PCT
P-LCR

Table S1. The blood routine test of ulcerative colitis patients.

Characteristics	Training set (n=307)	Internal validation set (n=244)	External validation set (n=424)
WBC (10^9/L)	7.60 (5.90-9.65)	7.10 (5.90-9.60)	7.39 (5.97-9.49)
NEUT% (%)	66.80 (58.20-72.75)	65.60 (58.95-73.95)	66.00 (57.65-73.22)
LYMPH% (%)	25.50 (18.95-32.95)	24.20 (16.75-30.30)	22.88 (16.50-31.62)
MONO% (%)	5.70 (4.50-7.35)	6.65 (4.90-8.50)	6.74 (5.40-8.52)
EO% (%)	1.40 (0.70-3.15)	1.70 (0.70-3.30)	1.80 (0.80-3.32)
BASO% (%)	0.00 (0.00-0.20)	0.10 (0.00-0.30)	0.30 (0.10-0.60)
NEUT (10^9/L)	4.88 (3.63-6.75)	4.71 (3.52-6.77)	4.76 (3.53-6.72)
LYMPH (10^9/L)	1.88 (1.44-2.43)	1.69 (1.24-2.09)	1.71 (1.32-2.09)
MONO (10^9/L)	0.45 (0.31-0.62)	0.45 (0.33-0.65)	0.50 (0.38-0.70)
EO (10^9/L)	0.12 (0.05-0.24)	0.13 (0.05-0.24)	0.13 (0.06-0.26)
BASO (10^9/L)	0.00 (0.00-0.01)	0.01 (0.00-0.02)	0.02 (0.01-0.05)
HGB (g/dl)	130 (112-142)	126 (108-140)	126 (111-141)
RBC (10^{12}/L)	4.42 (4.00-4.70)	4.36 (3.96-4.70)	4.38 (3.97-4.76)
HCT (%)	38.30 (34.20-41.35)	38.35 (33.80-41.70)	38.40 (34.00-42.43)
MCV (fl)	86.9 (83.1-90.4)	88.2 (84.2-91.4)	88.3 (84.3-92.2)
MCH (pg)	29.60 (27.65-30.90)	29.30 (27.45-30.50)	29.68 (27.80-30.80)
MCHC (g/L)	337 (327-344)	327 (317-337)	331 (321-341)
RDW-CV (%)	13.1 (12.6-13.9)	13.0 (12.4-14.2)	12.7 (12.1-13.6)
RDW-SD (fl)	41.60 (39.95-43.60)	41.60 (39.80-44.00)	40.90 (39.45-43.30)
PLT (10^9/L)	281 (227-369)	278 (223-368)	268 (218-365)
MPV (fl)	10.3 (9.6-11.0)	10.1 (9.4-11.0)	9.2 (8.2-10.2)
PDW (fl)	11.70 (10.60-13.20)	10.90 (9.95-12.30)	15.4 (11.7-16.2)
PCT (%)	0.29 (0.25-0.37)	0.30 (0.23-0.37)	0.25 (0.20-0.31)
P-LCR (%)	26.9 (21.4-32.8)	24.7 (19.7-30.6)	23.3 (18.0-28.7)

Table S2. Analysis of variance of complete blood count of ulcerative colitis patients in the training set.

Variables	*P*					
	E2 *vs.* E1	E3 *vs.* E1	Mayo score	Truelove & Witts score	Mayo endoscopic score	DUBLIN score
WBC (10^9/L)	**0.007**	**<0.001**	**<0.001**	**<0.001**	**0.006**	**<0.001**
NEUT% (%)	0.068	**0.003**	**<0.001**	**0.001**	**<0.001**	**<0.001**
LYMPH% (%)	**0.038**	**0.001**	**<0.001**	**<0.001**	**<0.001**	**<0.001**
MONO% (%)	0.711	0.786	0.55	0.405	0.065	0.859
EO% (%)	0.507	0.096	0.112	0.21	0.134	0.141
BASO% (%)	0.400	0.145	**<0.001**	0.053	**0.003**	0.108
NEUT (10^9/L)	**0.005**	**<0.001**	**<0.001**	**<0.001**	**<0.001**	**<0.001**
LYMPH (10^9/L)	0.449	0.582	0.233	0.988	0.175	0.878
MONO (10^9/L)	**0.032**	**0.001**	**<0.001**	**<0.001**	**<0.001**	**0.004**
EO (10^9/L)	0.188	**0.007**	0.686	0.636	0.249	0.531
BASO (10^9/L)	0.527	0.302	**<0.001**	0.126	**0.003**	0.194
HGB (g/dl)	**0.027**	**0.012**	**<0.001**	**<0.001**	**<0.001**	**<0.001**
RBC (10^{12}/L)	0.242	0.069	**<0.001**	**<0.001**	**0.005**	**0.001**
HCT (%)	0.078	0.026	**<0.001**	**<0.001**	**0.001**	**0.001**
MCV (fl)	0.051	0.255	**0.002**	**0.002**	0.222	0.339
MCH (pg)	**0.012**	0.128	**<0.001**	**<0.001**	**0.004**	0.068
MCHC (g/L)	0.056	0.051	**<0.001**	**0.009**	**0.003**	**0.005**
RDW-CV (%)	**<0.001**	**0.001**	**0.007**	0.043	**0.049**	**0.008**
RDW-SD (fl)	0.019	0.072	0.325	0.727	0.112	0.115
PLT (10^9/L)	**0.002**	**<0.001**	**<0.001**	**<0.001**	**0.013**	**<0.001**
MPV (fl)	0.259	**0.035**	**0.006**	**<0.001**	**0.026**	**0.004**
PDW (fl)	0.131	**0.016**	**0.011**	**<0.001**	**0.018**	**0.003**
PCT (%)	**0.001**	**<0.001**	**<0.001**	**<0.001**	**0.026**	**<0.001**
P-LCR (%)	0.197	**0.013**	**0.007**	**<0.001**	**0.029**	**0.008**

E2 vs. E1 presents distinguish left-sided from proctitis, E3 vs. E1 presents distinguish extensive from proctitis. Abbreviations: BASO, basophil; CV, coefficient of variation; DUBLIN, Degree of Ulcerative colitis Burden of Luminal Inflammation; EO, eosinophil; HCT, hematocrit; HGB, hemoglobin; IQR, interquartile range; LYMPH, lymphocyte; MCH, mean corpuscular hemoglobin; MCHC, mean corpuscular hemoglobin concentration; MCV, mean corpuscular volume; MONO, monocyte; MPV, mean platelet volume; NEUT, neutrophil; NLR, negative likelihood ratio; NPV, negative predictive value; PCT, thrombocytocrit; PDW, platelet distribution width; P-LCR, platelet large cell ratio; PLR, positive likelihood ratio; PLT, platelet; PPV, positive predictive value; RBC, red blood cell; RDW, red cell distribution width; SD, standard deviation; WBC, white blood cell.

Table S3. Jin's model adjusted for gender and age.

Model		P value	OR	0.95 CI
E2 vs E1	Gender	0.316	1.557	0.656-3.698
	Age	0.960	0.999	0.964-1.035
	Prediction value	0.004	125.536	4.834-3260.145
E3 vs E1	Gender	0.157	1.791	0.798-4.019
	Age	0.949	0.999	0.966-1.032
	Prediction value	<0.001	1760.341	88.238-35118.847
Mayo score	Gender	0.963	1.007	0.756-1.340
	Age	0.969	1.000	0.989-1.011
	Prediction value	<0.001	105.442	49.357-225.261
Truelove & Witts score	Gender	0.477	0.814	0.463-1.434
	Age	0.149	0.984	0.962-1.006
	Prediction value	<0.001	137.688	15.800-1199.889
Mayo endoscopic score	Gender	0.488	0.805	0.435-1.488
	Age	0.191	1.015	0.993-1.038
	Prediction value	<0.001	62.167	9.196-420.272
DUBLIN score	Gender	0.945	0.981	0.570-1.688
	Age	0.213	1.014	0.992-1.035
	Prediction value	<0.001	281.031	30.12-2631.538

E2 vs. E1 presents distinguish left-sided from proctitis, E3 vs. E1 presents distinguish extensive from proctitis. Abbreviations: CI, confidence interval; DUBLIN, Degree of Ulcerative colitis Burden of Luminal Inflammation; OR, odds ratio.

Table S4. The illustration of Jin's model for predicting UC extent.

<table>
<tr><th colspan="2">Results Classification</th><th>Result 1</th><th>Result 2</th><th>Result 3</th><th>Result 4</th></tr>
<tr><td rowspan="2">Model</td><td>Left-sided</td><td>(+)</td><td>(+)</td><td>(-)</td><td>(-)</td></tr>
<tr><td>Proctitis</td><td>(-)</td><td>(-)</td><td>(+)</td><td>(+)</td></tr>
<tr><td rowspan="2">Model</td><td>Extensive</td><td>(+)</td><td>(-)</td><td>(+)</td><td>(-)</td></tr>
<tr><td>Proctitis</td><td>(-)</td><td>(+)</td><td>(-)</td><td>(+)</td></tr>
<tr><td colspan="2" rowspan="2">Interpretation of the results</td><td>Left-sided> Proctitis</td><td rowspan="2">Left-sided> Proctitis> Extensive</td><td rowspan="2">Extensive> Proctitis > Left-sided</td><td>Proctitis> Left-sided</td></tr>
<tr><td>Extensive> Proctitis</td><td>Proctitis> Extensive</td></tr>
<tr><td colspan="2">Output</td><td>Left-sided / Extensive</td><td>Left-sided</td><td>Extensive</td><td>Proctitis</td></tr>
</table>

Table S5. Univariate and multivariate analysis of independent factors in Jin's model for differentiating left-sided UC form proctitis UC.

	Univariate analysis			Training set			Internal validation set			External validation set		
	OR	0.95 CI	P value	OR	0.95 CI	Adjusted P	OR	0.95 CI	Adjusted P	OR	0.95 CI	Adjusted P
Gender						0.293			0.434			0.853
Age						0.966			0.449			0.401
WBC	1.299	1.077-1.568	0.006	1.260	1.040-1.528	0.018	1.310	1.061-1.617	0.012	1.711	1.376-2.128	<0.001
RDW-CV	1.632	1.053-2.528	0.028	1.620	1.044-2.514	0.031	1.481	1.012-2.168	0.043	2.219	1.486-3.314	<0.001
PCT	2682.988	9,864-729731.787	0.006									

Abbreviations: CI, confidence interval; CV, coefficient of variation; OR, odds ratio; PCT, thrombocytocrit; RBC, red blood cell; RDW, red cell distribution width; WBC, white blood cell.

Table S6. Univariate and multivariate analysis of independent factors in Jin's model for differentiating extensive UC form proctitis UC.

	Univariate analysis			Training set			Internal validation set			External validation set		
	OR	0.95 CI	P value	OR	0.95 CI	Adjusted P	OR	0.95 CI	Adjusted P	OR	0.95 CI	Adjusted P
Gender						0.137			0.188			0.827
Age						0.944			0.247			0.126
LYMPH%	0.935	0.898-0.973	0.001	0.943	0.904-0.984	0.007	0.933	0.888-0.981	0.007	0.922	0.890-0.955	<0.001
EO	57.788	1.998-1671.593	0.018			0.059	82.632	1.644-4152.692	0.027	395.343	18.531-8434.143	<0.001
PLT	1.009	1.004-1.013	< 0.001	1.009	1.004-1.015	0.001	1.018	1.009-1.026	<0.001	1.006	1.002–1.010	0.005

Abbreviations: CI, confidence interval; CV, coefficient of variation; EO, eosinophil; LYMPH, lymphocyte; OR, odds ratio; PLT, platelet.

Table S7. Univariate and multivariate analysis of independent factors in Jin’s model for Mayo score.

		Univariate analysis			Training set			Internal validation set			External validation set		
		OR	0.95 CI	P value	OR	0.95 CI	Adjusted P	OR	0.95 CI	Adjusted P	OR	0.95 CI	Adjusted P
Gender	Remission/mild												
	Moderate												
	Female							1					
	Male						0.515	2.064	1.063-4.010	0.032			0.245
	Severe												
	Female												
	Male						0.204			0.426			0.406
Age	Remission/mild												
	Moderate						0.957			0.346			0.099
	Severe						0.991			0.233			0.09
WBC	Remission/mild	1											
	Moderate	1.124	1.028-1.229	0.011	1.144	1.039-1.260	0.006	1.175	1.046-1.319	0.006	1.364	1.196-1.566	< 0.001
	Severe	1.304	1.162-1.464	<0.001	1.351	1.189-1.536	<0.001	1.571	1.321-1.869	<0.001	1.446	1.253-1.669	<0.001
NEUT%	Remission/mild	1											
	Moderate	1.039	1.014-1.065	0.002									
	Severe	1.071	1.030-1.114	<0.001									
LYMPH%	Remission/mild	1											
	Moderate	0.958	0.933-0.983	0.001									
	Severe	0.925	0.886-0.965	<0.001									
MONO%	Remission/mild												
	Moderate			0.546									
	Severe			0.118									
EO%	Remission/mild												
	Moderate			0.758									
	Severe			0.217									
BASO%	Remission/mild	1											
	Moderate	0.045	0.009-0.233	<0.001									
	Severe	0.059	0.004-0.848	0.037									
NEUT	Remission/mild	1											
	Moderate	1.148	1.035-1.274	0.009									

	Severe	1.324	1.162-1.507	<0.001									
LYMPH	Remission/mild												
	Moderate			0.228									
	Severe			0.583									
MONO	Remission/mild	1											
	Moderate	4.119	1.328-12.773	0.014									
	Severe	19.255	4.810-77.070	<0.001									
EO	Remission/mild												
	Moderate			0.408									
	Severe			0.928									
BASO	Remission/mild												
	Moderate			0.002									
	Severe			0.147									
HGB	Remission/mild	1											
	Moderate	0.974	0.961-0.986	<0.001									
	Severe	0.952	0.935-0.968	<0.001									
RBC	Remission/mild	1											
	Moderate	0.541	0.349-0.837	0.006									
	Severe	0.262	0.141-0.488	<0.001									
HCT	Remission/mild	1											
	Moderate	0.912	0.868-0.958	<0.001	0.898	0.847-0.951	<0.001	0.875	0.820-0.934	<0.001	0.944	0.898-0.993	0.026
	Severe	0.828	0.774-0.885	<0.001	0.792	0.731-0.858	<0.001	0.725	0.646-0.813	<0.001	0.86	0.810-0.914	<0.001
MCV	Remission/mild	1											
	Moderate	0.958	0.922-0.996	0.029									
	Severe	0.905	0.861-0.951	<0.001									
MCH	Remission/mild	1											
	Moderate	0.872	0.797-0.953	0.002									
	Severe	0.781	0.699-0.873	<0.001									
MCHC	Remission/mild	1											
	Moderate	0.975	0.960-0.990	0.001									
	Severe	0.96	0.941-0.980	<0.001									
RDW-CV	Remission/mild	1											
	Moderate	1.228	1.022-1.476	0.029									
	Severe	1.501	1.221-1.846	<0.001									

RDW-SD	Remission/mild			
	Moderate			0.773
	Severe			0.153
PLT	Remission/mild	1		
	Moderate	1.003	1.000-1.005	0.043
	Severe	1.007	1.003-1.010	<0.001
MPV	Remission/mild			
	Moderate			0.351
	Severe			0.003
PDW	Remission/mild			
	Moderate			0.407
	Severe			0.014
PCT	Remission/mild	1		
	Moderate	19.789	1.385-282.704	0.028
	Severe	1223.893	39.548-37876.261	<0.001
P-LCR	Remission/mild			
	Moderate			0.279
	Severe			

Abbreviations: BASO, basophil; CI, confidence interval; CV, coefficient of variation; EO, eosinophil; HCT, hematocrit; HGB, hemoglobin; LYMPH, lymphocyte; MCH, mean corpuscular hemoglobin; MCHC, mean corpuscular hemoglobin concentration; MCV, mean corpuscular volume; MONO, monocyte; MPV, mean platelet volume; NEUT, neutrophil; OR, odds ratio; PCT, thrombocytocrit; PDW, platelet distribution width; P-LCR, platelet large cell ratio; PLT, platelet; RBC, red blood cell; RDW, red cell distribution width; SD, standard deviation; WBC, white blood cell.

Table S8. Univariate and multivariate analysis of independent factors in Jin's model for Mayo endoscopic score.

	Univariate analysis			Training set			Internal validation set			External validation set		
	OR	0.95 CI	P value	OR	0.95 CI	Adjusted P	OR	0.95 CI	Adjusted P	OR	0.95 CI	Adjusted P
Gender						0.185			0.074			0.211
Age						0.059			0.654			0.636
WBC			0.066									
NEUT%	1.028	1.003-1.054	0.029									
LYMPH%	0.973	0.947-0.999	0.039									
BASO%			0.075									
NEUT	1.138	1.024-1.264	0.017	1.159	1.037-1.296	0.009			0.14	1.2	1.023-1.047	0.025
MONO			0.068									
BASO			0.135									
HGB	0.981	0.969-0.993	0.002									
RBC	0.507	0.323-0.798	0.003									
HCT	0.929	0.985-0.975	0.003	0.944	0.894-0.996	0.035	0.902	0.847-0.960	0.001	0.904	0.843-0.971	0.005
MCH			0.216									
MCHC	0.984	0.970-0.999	0.039									
PLT			0.051									
MPV			0.057									
PDW			0.067									
PCT			0.077									

Abbreviations: BASO, basophil; CI, confidence interval; HCT, hematocrit; HGB, hemoglobin; LYMPH, lymphocyte; MCH, mean corpuscular hemoglobin; MCHC, mean corpuscular hemoglobin concentration; MONO, monocyte; MPV, mean platelet volume; NEUT, neutrophil; OR, odds ratio; PCT, thrombocytocrit; PDW, platelet distribution width; PLT, platelet; RBC, red blood cell; WBC, white blood cell.

Table S9. Univariate and multivariate analysis of independent factors in Jin's model for DUBLIN score.

	Univariate analysis			Training set			Internal validation set			External validation set		
	OR	0.95 CI	P value	OR	0.95 CI	Adjusted P	OR	0.95 CI	Adjusted P	OR	0.95 CI	Adjusted P
Gender						0.798			0.139			0.951
Age						0.359			0.324			0.090
WBC	1.158	1.061-1.264	0.001	1.203	1.092-1.325	<0.001	1.149	1.039-1.271	0.007	1.668	1.384-2.011	<0.001
RBC	0.466	0.030-0.716	<0.001	0.428	0.258-0.708	<0.001	0.274	0.146-0.512	<0.001	0.437	0.249-0.765	0.004
MCHC	0.980	0.966-0.994	0.006									
RDW-CV	1.220	1.034-1.440	0.019									

Abbreviations: CI, confidence interval; CV, coefficient of variation; MCHC, mean corpuscular hemoglobin concentration; OR, odds ratio; RBC, red blood cell; RDW, red cell distribution width; WBC, white blood cell.

Table S10. Summary evaluation of prediction model for Mayo score with each method.

		Accuracy	Sensitivity	Specificity	PPV	NPV	PLR	NLR	F1-Score
Logistic regression	Train	0.73	0.59	0.80	0.59	0.80	2.91	0.51	0.68
	Internal validation	0.70	0.55	0.78	0.55	0.78	2.48	0.58	0.65
	External validation	0.71	0.57	0.78	0.57	0.78	2.63	0.55	0.66
Adaboost	Train	0.71	0.56	0.79	0.58	0.78	2.67	0.56	0.67
	Internal validation	0.66	0.49	0.74	0.49	0.74	1.90	0.69	0.59
	External validation	0.64	0.46	0.73	0.46	0.73	1.67	0.75	0.56
Random forest	Train	1.00	1.00	1.00	1.00	1.00	-	0.00	1.00
	Internal validation	0.70	0.55	0.77	0.55	0.77	2.43	0.58	0.64
	External validation	0.69	0.54	0.77	0.54	0.77	2.30	0.61	0.63
Decision tree	Train	1.00	1.00	1.00	1.00	1.00	-	0.00	1.00
	Internal validation	0.67	0.50	0.75	0.50	0.75	2.03	0.66	0.60
	External validation	0.64	0.47	0.73	0.47	0.73	1.75	0.73	0.57

Abbreviations: NLR, negative likelihood ratio; NPV, negative predictive value; PLR, positive likelihood ratio; PPV, positive predictive value.

APPENDIX

Appendix 1. The details of the construction and evaluation of model

1.1 Multinomial regression with elastic-net penalty

Multinomial logit model: $X = (x_1, x_2, .., x_n)$ is the independent variables for K possible outcomes. We running $K - 1$ independent binary logistic regression model in which $K - th$ outcome is chosen as a "pivot" and then the other $K - 1$ outcomes are separately regressed again it. $\beta_1, .., \beta_{K-1}$ are coefficients for each binary regression. The $K - 1$ binary regression models are as follows.

$$\ln \frac{P(Y_i = 1)}{P(Y_i = K)} = \beta_1 \cdot X_i$$

$$ln \frac{P(Y_i = 2)}{P(Y_i = K)} = \beta_2 \cdot X_i$$

$$\dots\dots$$

$$\ln \frac{P(Y_i = K - 1)}{P(Y_i = K)} = \beta_{K-1} \cdot X_i$$

$$\ln \frac{P(Y_i = K - 1)}{P(Y_i = K)} = \beta_{K-1} \cdot X_i$$

When doing predictions, the class with the largest predicted probability is chosen.

$$P(Y_i = 1) = \frac{e^{\beta_1 \cdot X_i}}{\sum_{k=1}^{K} e^{\beta_k \cdot X_i}}$$

$$P(Y_i = 2) = \frac{e^{\beta_2 \cdot X_i}}{\sum_{k=1}^{K} e^{\beta_k \cdot X_i}}$$

$$\dots\dots$$

$$P(Y_i = K) = \frac{e^{\beta_K \cdot X_i}}{\sum_{k=1}^{K} e^{\beta_k \cdot X_i}}$$

1.1.1 Elastic-net penalty:

l_1 regularization used in Lasso regression can select variables. L_2 regression used in ridge regression can reduce estimation variance when there is collinearity between variables. Elastic-Net penalty linearly combined the two penalties and has the form as $\lambda\left(\alpha||\beta||_1 + (1 - \alpha)||\beta||_2\right)$.

The loss function is the sum of Elastic-Net Penalty and log-likelihood function, which is

$$\sum_i \sum_k \mathbf{1}\,(Y_i = k)P(Y_i = k) + \lambda(\alpha \sum_k ||\beta_k||_1 + (1 - \alpha)\sum||\beta_k||_2)$$

1.1.2 Five-folds cross validation:

We select the value of hyper-parameters λ and α by five-folds cross validation. The original sample is randomly partitioned into five equal sized subsamples. Of the five subsamples, a single subsample is retained as the validation data for testing the model, and the remaining subsamples are used as training data. The cross-validation process is then repeated five times, with each of the five subsamples used exactly once as the validation data. The five results can then be averaged to evaluate the model performance.

1.2 Non-linear transformation

In regression analysis, when a residual plot reveals a data set to be nonlinear, analysts sometimes apply nonlinear transformations to the independent and/or dependent variables. If this transformation increases the linearity of the relationship between the variables, it allows the analyst to use linear regression techniques appropriately with nonlinear data.

In our experiment, we implement different non-linear transformations to our variables, including polynomial transformation, reciprocal transformation, and logarithmic transformation. Then we select the transformation with the best prediction performance.

Suppose X is independent variable. Polynomial transformation adds X^k (k is polynomial degree) to independent variables. Reciprocal transformation adds $\frac{1}{X}$ to independent variables. Logarithmic transformation adds $\log X$ to independent variables.

1.3 Interaction effects

In regression, an interaction effect exists when the effect of an independent variable on a dependent variable change, depending on the value(s) of one or more other independent variables.

In a regression equation, an interaction effect is represented as the product of two or more independent variables. For example, here is a typical regression equation without an interaction is $Y = \beta_0 + \beta_1 X_1 + \beta_2 X_2 + \varepsilon$ where Y is dependent variables and X_I is independent variables.

And here is the same regression equation with an interaction:$Y = \beta_0 + \beta_1 X_1 + \beta_2 X_2 + \beta_3 X_1 X_2 + \varepsilon$ Here, β_3 is a regression coefficient, and $X_1 X_2$ is the interaction.

1.4 Class-weighted loss function

For Mayo score, Mayo endoscopic score (MES) and Truelove & Witts score (TWS), there are severe class-imbalance in derivation cohort. This will make the prediction results biased towards the majority class. We use class-weighted loss to reduce this bias. $n_1,..,n_K$ denote the sample size of each class. We multiply the loss of each sample by $\frac{1}{n_k}$. The class-weighted loss function is

$$\sum_i \sum_k \frac{1}{n_k} \mathbf{1}(Y_i = k) P(Y_i = k) + \lambda\,(\alpha \sum_k ||\beta_k||_1 + (1 - \alpha)\sum||\beta_k||_2)$$

1.5 Micro-average area under the receiver operating characteristic (AUROC) curve

Micro-average AUROC is used to evaluate the performance of multi-classification model. N is the sample size. K is the number of possible outcomes. L is a $N \times K$ matrix where the $i-th$ line and $k-th$ column element is the ground truth $\mathbf{1}(Y_i = k)$. P is a $N \times K$ matrix where the $i-th$. line and $k-th$ column element is the predicted probability $P(Y_i = k)$. We expand matrix L and P by rows and form two columns of length $m \times n$ after transposing. In this way, the multi-category outcome can be converted into a two-category situation, and then the classic two-category outcome ROC analysis will be carried out and the AUROC can be calculated. This AUC is called micro-average AUROC.

Appendix 2. The description of Jin's model

2.1 The model for predicting extent

2.1.1 The model for differentiating left-sided UC form proctitis UC

$$risk = \frac{e^z}{1 + e^z}$$

Where

$$z = 0.216793872866383 * \mathrm{WBC} - 0.0147954956920771 * \mathrm{RDWCV} + 6.22633232763057 * \mathrm{PCT} - 2.22144115362666$$

2.1.2 The model for differentiating extensive UC form proctitis UC

$$risk = \frac{e^z}{1 + e^z}$$

Where

$$\mathrm{z} = -0.0576486021133527 * LYMPH\% + 3.98849607881532 * EO + 0.00840662741273303 * PLT + 0.530022062742962$$

2.2 The model for predicting severity

2.2.1 The model for predicting Mayo score

$$risk_{Remission} = \frac{risk_1}{risk_1 + risk_2 + risk_3 + risk_4}$$

$$risk_{Mild} = \frac{risk_2}{risk_1 + risk_2 + risk_3 + risk_4}$$

$$risk_{Moderate} = \frac{risk_3}{risk_1 + risk_2 + risk_3 + risk_4}$$

$$risk_{Severe} = \frac{risk_4}{risk_1 + risk_2 + risk_3 + risk_4}$$

$$risk_1 = e^{Z_1}$$

$$risk_2 = e^{Z_2}$$

$$risk_3 = e^{Z_3}$$

$$risk_4 = e^{Z_4}$$

$$z_1 = -6.911414087217679E^{-5} * \mathrm{NEUT\%} * \mathrm{MCHC} - 7.368970062736082E^{-5} * \mathrm{NEUT\%} * \mathrm{PLT} + 4.1554479272002994E^{-5} * \mathrm{HGB} * \mathrm{MCHC} + 1.52630682515538E^{-5} * \mathrm{HGB} * \mathrm{PLT} - 5.631670538601904E^{-6} * \mathrm{MCV} * \mathrm{MCHC} - 1.2583168411254944E^{-5} * \mathrm{MCV} * \mathrm{PLT} + 3.4439060671920207E^{-7} * \mathrm{MCHC} * \mathrm{PLT}$$

$$z_2 = -2.999579759892875E^{-5} * \text{NEUT\%} * \text{MCHC} + 4.330526035643579E^{-6} * \text{HGB} * \text{MCV} + 3.349735180592343E^{-5} * \text{HGB} * \text{MCHC} + 4.277969717054117E^{-5} * \text{MCV} * \text{PLT} - 1.0407203593932822E^{-5} * \text{MCHC} * \text{PLT} + 3.24586685636925E^{-5} * \text{MCHC} * \text{MPV} + 2.2563527272048465E^{-5} * \text{PLT} * \text{MPV} - 2.416023383154776E^{-6} * \text{PLT} * PLCR$$

$$z_3 = -3.645534694569229E^{-6} * \text{WBC} * \text{PLT} + 5.1896825692123295E^{-5} * \text{NEUT\%} * \text{MCHC} + 2.57442985448E^{-5} * \text{NEUT\%} * \text{PLT} - 2.3211542959545734E^{-5} * \text{HGB} * \text{MCHC} - 3.389164878990511E^{-6} * \text{HGB} * \text{PLT} + 2.5469244129487984E^{-5} * \text{MCV} * \text{MCHC} + 4.670465034899894E^{-10} * \text{MCHC} * \text{PLT} - 5.631516525644669E^{-6} * \text{MCHC} * \text{MPV} + 1.6642070378144477E^{-6} * \text{MCHC} * \text{PLCR} - 5.682685716770145E^{-7} * \text{PLT} * \text{MPV} + 1.3629383424438393E^{-5} * \text{PLT} * \text{PLCR}$$

$$z_4 = 2.858593958984906E^{-5} * \text{WBC} * \text{MCHC} + 3.063338507769241E^{-5} * \text{WBC} * \text{PLT} + 4.9218617667251204E^{-5} * \text{NEUT\%} * \text{MCHC} + 3.981583325375622E^{-5} * \text{NEUT\%} * \text{PLT} + 1.5374763837362375E^{-5} * \text{NEUT} * \text{MCHC} + 1.48287628107446E^{-5} * \text{NEUT} * \text{PLT} - 6.680058280726672E^{-7} * \text{HGB} * \text{MCV} - 5.292384393386941E^{-5} * \text{HGB} * \text{MCHC} - 3.1645862438216435E^{-6} * \text{HGB} * \text{PLT} - 9.275820513549848E^{-7} * \text{HCT} * \text{MCHC} - 1.294850078819621E^{-10} * \text{MCV} * \text{MCHC} - 7.249789479892556E^{-6} * \text{MCV} * \text{PLT} + 4.8445452277448145E^{-6} * \text{MCHC} * \text{PLT} - 1.1856164809495403E^{-5} * \text{MCHC} * \text{PLCR}$$

2.2.2 The model for predicting Truelove & Witts score

$$risk_{Mild} = \frac{risk_1}{risk_1 + risk_2 + risk_3}$$

$$risk_{Moderate} = \frac{risk_2}{risk_1 + risk_2 + risk_3}$$

$$risk_{Severe} = \frac{risk_3}{risk_1 + risk_2 + risk_3}$$

$$risk_1 = e^{Z_1}$$

$$risk_2 = e^{Z_2}$$

$$risk_3 = e^{Z_3}$$

$$z_1 = -0.04613 * \text{WBC} + 0.00615 * \text{NEUT\%} + 0.03222 * \text{LYMPH\%} - 0.01519 * \text{NEUT} - 0.00017 * \text{MONO} + 0.00723 * \text{HGB} - 0.00032 * \text{RBC} - 0.01249 * \text{HCT} + 0.00392 * \text{MCV} + 0.00252 * \text{MCH} - 0.00133 * \text{MCHC} - 0.00039 * \text{PLT} - 0.00885 * \text{MPV} - 0.00027 * \text{PDW} + 0.00168 * \text{PCT}$$

$$z_2 = 0.00214 * \text{WBC} - 0.00027 * \text{NEUT\%} - 0.00031 * \text{LYMPH\%} + 0.00023 * \text{NEUT} - 0.00095 * \text{HGB} + 0.00789 * \text{HCT} + 0.01119 * \text{MCV} - 0.00011 * \text{MCH} - 0.00171 * \text{MCHC} - 0.00008 * \text{PLT} - 0.00079 * \text{MPV} - 0.00095 * \text{PDW} - 0.00147 * \text{PCT} + 0.01755 * \text{PLCR}$$

$$z_3 = 0.03277 * \text{WBC} - 0.00255 * \text{NEUT\%} - 0.00722 * \text{LYMPH\%} + 0.01042 * \text{NEUT} - 0.02696 * \text{HGB} + 0.00251 * \text{HCT} - 0.02387 * \text{MCV} - 0.00441 * \text{MCH} + 0.01817 * \text{MCHC} + 0.00246 * \text{PLT} + 0.00661 * \text{MPV} + 0.00027 * \text{PDW} - 0.02461 * \text{PLCR}$$

2.2.3 The model for predicting Mayo endoscopic score

$$risk = \frac{e^z}{1 + e^z}$$

$$\text{z} = -0.012237568731133132 * \text{WBC} + 0.052529720493550015 * \text{NEUT\%} + 0.04727080881527114 * \text{LYMPH\%} - 0.031718855841914986 * \text{BASO\%} + 0.1330681876159437 * \text{NEUT} + 0.04823754878543689 * \text{MONO} - 0.0030646780080299243 * \text{BASO} - 0.015520270944795368 * \text{HGB} + 0.00877681334369144 * \text{RBC} - 0.03410645879851377 * \text{HCT} + 0.1653720481640234 * \text{MCH} - 0.01903679823526698 * \text{MCHC} - 9.423727885764605E^{-5} * \text{PLT} - 0.057187399852513116 * \text{MPV} + 0.01637019638470416 * \text{PDW} - 0.011655144919472733 * \text{PCT}$$

2.2.4 The model for predicting DUBLIN score

$$risk = \frac{e^z}{1 + e^z}$$

Where

$$z = 0.182332514951181 * \mathrm{WBC} - 0.792766558900402 * \mathrm{RBC} - 0.0151163230904716 * \mathrm{MCHC} - 0.0302617731208481 * \mathrm{RDWCV} + 8.28789687234169$$
